# The high normal ankle brachial index is associated with left ventricular hypertrophy in hypertension patients among the Han Chinese

**DOI:** 10.1111/jch.14328

**Published:** 2021-07-23

**Authors:** Jin Sun, Shuxia Wang, Man Li, Yongkang Su, Shouyuan Ma, Yan Zhang, Anhang Zhang, Shuang Cai, Bokai Cheng, Qiligeer Bao, Ping Zhu

**Affiliations:** ^1^ Medical School of Chinese PLA Beijing China; ^2^ Department of Geriatrics The second Medical Center & National Clinical Research Center for Geriatric Diseases Chinese PLA General Hospital Beijing China; ^3^ Department of Geriatric Cardiology The second Medical Center, Chinese PLA General Hospital Beijing China; ^4^ Department of Outpatient The first Medical Center, Chinese PLA General Hospital Beijing China

**Keywords:** ankle brachial index, echocardiography, hypertension, left ventricular hypertrophy

## Abstract

Left ventricular hypertrophy (LVH) is the most common target organs damage in the hypertension patients. Abnormal low (≤0.9) or high (≥1.40) ankle brachial index (ABI) are associated with an increased risk of cardiovascular events. However, the relationships between a high ABI in the normal range (0.9–1.4) and LVH in Han Chinese hypertension are not entirely elucidated. This study included 3953 hypertension patients aged 40–75 years among Han Chinese. Hypertension was defined as systolic blood pressure≥140 mm Hg, diastolic blood pressure≥90 mm Hg, or history of antihypertensive drug use. Left ventricular mass (LVM) was measured by transthoracic echocardiography. LVH was diagnosed by using the criteria of LVM ≥49.2 g/m^2.7^ for men and 46.7 g/m^2.7^ for women. Our study suggested that the ABI was higher in patients with LVH than in those without (1.13±0.11, 1.11±0.11, *p* < 0.001). The prevalence of LVH in patients with the lowest (0.9 < ABI≤1.03), second (1.04≤ABI≤1.11), the third (1.12≤ABI≤1.20), and the highest quartile (1.21≤ABI < 1.40) of ABI was 37.2%, 38.2%, 45.5%, 45.7%, respectively. Logistic regression analysis suggested that the highest and third quartile of ABI were significantly associated with increased LVH risk (multivariate‐adjusted OR of highest group:1.83; third group:1.61). The association of ABI at second quartile with LVH was nonsignificant. Similar results were observed in less than 60 years and without coronary heart disease or diabetes group. Our observations in Chinese patients with hypertension indicated high ABI may be an important risk factor for LVH in hypertension patients among Han Chinese, even in the normal range.

## 1 INTRODUCTION

Ankle brachial index (ABI) is the ratio of the systolic blood pressure (SBP) in the ankle divided by SBP in the brachial artery, which is the best non‐invasive index for the diagnosis of flow‐limiting lower extremity peripheral arterial disease (PAD).^1^ Several studies have shown that ABI serves as a measure of systemic atherosclerosis, not just in the leg arteries, and is associated with both cardiovascular risk factors and prevalent cardiovascular event.^2^ Several large population‐based cohort studies have shown that ABI≤0.9 is an independent predictor of coronary heart disease (CAD), myocardial infarction, stroke, ejection fraction‐preserved heart failure, and mortality.^3^, ^4^, ^5^, ^6^ ABI might improve the accuracy of risk prediction scoring systems. Abnormally high ABI were also associated with increased risk of cardiovascular events in different population.^7^, ^8^ A study of native Americans living in the community reported that a U‐shaped relationship between ABI levels and cardiovascular events, that is, patients with an abnormally high or low ABI had about twice the risk of developing cardiovascular disease compared with those with a normal ABI.^5^

ABI is also associated with many cardiovascular risk factors, including hypertension and secondary target organ damage.^9^, ^10^ LVH is the most common complication of hypertension. Joachim and coworkers demonstrated the association between abnormally high ABI and increased left ventricular mass (LVM) by cardiac magnetic resonance imaging (MRI) in the Multi‐ethnic Atherosclerosis Study (MESA).^11^ Abnormally high ABI is associated with calcification of the medial arteries and, therefore, predicts the development of LVH, since artery calcification and increased stiffness lead to increased left ventricular afterload, directly leading to LVH. This result has been confirmed in several subsequent studies.^12^ In addition, abnormally low ABI is independently associated with increased LVM in people with hypertension or chronic kidney disease.^13^, ^14^ Recently, Hidekazu and co‐workers found that a high ABI in the normal range was significantly associated with ECG‐determined LVH in a cross‐sectional study of Japanese community populations.^15^ However, the relationship between high ABI within the normal range and LVH in patients with hypertension is still unknown. Therefore, the objective of this study was to investigate the relationship between a high normal ABI and LVH in the hypertensive Chinese population.

## 2 METHODS

### 2.1 Participants

This was a community‐based cross‐sectional study, and participants were recruited from the Xinyang County, in the middle region in China from 2004 to 2005. We used a multistage cluster sample method to select a representative sample of rural community residents aged 40–75 years. A total of 13,444 patients (5270 men and 8174 women) incorporated into the survey, which were from 63 district of Xinyang's seven residential communities and yielding a response rate of 84.9%. Among them, 5421 hypertensive patients were identified and thoroughly examined. Hypertension was defined as diastolic blood pressure (DBP) of ≥90 mm Hg, SBP of ≥140 mm Hg, physician diagnosis or current medication for hypertension (as defined by WHO 1999). Of 5421 hypertensive patients, 4805 patients had measured LVM through echocardiography, and 4460 patients underwent optimal voluntarily ABI measurements. The patients with ABI ≤ 0.9 (*n* = 386) or ABI ≥ 1.4 (*n* = 121) were excluded, because ABI ≤ 0.9 indicates PAD and ABI ≥ 1.4 was in accordance with arterial stiffness and wave reflection, including these values could lead to misclassification and interfere with statistical results. Ultimately, 3953 patients (1363 men and 2590 women) with integrated clinical and echocardiography data were remained in the present study.

The study protocol was reviewed and approved by the ethical committees of the Fuwai Hospital and local hospitals. All participants gave their informed consent before they were recruited and reported themselves to be Han people.

### 2.2 Ankle brachial index measurement

The ABI was measured using standard official protocol. SBP was measured twice using the portable Doppler device (ES‐101EX, HADECO, 8 MHz probe, Kawasaki, Japan) and standard 12‐cm cuff in each arm with the patients resting for at least 5 min. The Doppler Stethoscope was placed at the humeral artery fluctuation, quickly inflate the cuff to 20–30 mm Hg above the palpated SBP, and then deflated at the rate of 2–6 mm Hg/s. The first sound heard is the systolic pressure of the brachial artery. The cuff was then placed over the posterior tibial artery to measure ankle SBP of each leg.

The ABI was defined as the ankle SBP/ brachial SBP ratio. In present study, the ABI of each leg was calculated as the SBP in the posterior tibial artery divided by the higher of the two arms SBPs. The reason for using higher arm SBP was the strong association between PAD and subclavian artery stenosis. Patients‐specific ABIs were defined as the higher of their two legs ABI because of the lower value of ABI may represent arterial stenosis. Patients were divided into four groups according to ABI quartile: quartile 1 (0.9 < ABI≤1.03), quartile 2 (1.04≤ABI≤1.11), quartile 3 (1.12≤ABI≤1.20), and quartile 4 (1.20≤ABI < 1.40).

### 2.3 Echocardiographic methods

Transthoracic echocardiography was performed according to standard protocols, under the supervision of two ultrasound physicians with at least 2 years of experience, and performed by two technicians trained in echocardiography at the Institute of Cardiology, Chinese Academy of Medical Sciences. HP 5500 (Phillips health care system, Boston, MA, USA) or HDI 3000 (ATL, Bothell, WA, USA) was used to carry on M‐mode, two‐dimensional (2D) and color doppler records from the parasternal long axis and short axis windows, and 2D and color Doppler evaluations from the apical window to yield 2‐, 3‐, and 4 chamber images. The transducer frequency was 2.5–3.5 MHz. Patients who were unable to reach the local study center were screened using the Optigo echocardiographic recorders (Agilent, Boston, MA, USA).

As recommended by the American Society of Echocardiography, the echocardiographic measurements were made after the correct orientation of the two‐dimensional and Doppler imaging planes was confirmed using standard procedures, with the patients respiring quietly in the left decubitus position. The echocardiographic indicators were measured at the end of systolic and end diastolic periods of up to three cardiac cycles, including left atrium diameter, diastolic left ventricular inner diameter (LVIDD), diastolic left ventricular posterior wall thickness (PWTd), diastolic interventricular septal thickness (IVSd), E wave deceleration time, transmitted E wave velocity, and transmitted A wave velocity.

### 2.4 Calculation of derived variables

LVM was calculated by using the equation: 0.8×1.04 ((IVSd + LVIDD + PWTd)^3^—LVIDD^3^) + 0.6. LVMI was calculated by dividing LVM by height^2.7^ (LVMI_h2.7_). Relative wall thickness (RWT) was calculated using Devereux method, that is, 2×PWTd / LVIDD. LVH was diagnosed by using the criteria of LVMI more than 49.2 g/m^2.7^ for men and 46.7 g/m^2.7^ for women. Body surface area (BSA) was calculated by using the Stevenson formula: BSA (m^2^) = 0.0061 × height (cm) + 0.0128 × weight (kg) ‐ 0.1529. Body mass index (BMI) was calculated as the ratio of the weight in kg divided by the square of the height in m.

### 2.5 Statistical analysis

Data management and statistical analysis was performed using SPSS 22.0 for Windows (SPSS Inc, Chicago, IL, USA). Data are reported as the mean ± standard deviation for continuous variables and as percentages for categorical variables. Continuous variable independent sample t‐test and classified variable chi‐square test were used for the differences between LVH group and non‐LVH group. All participants were stratified by quartiles of ABI, baseline differences in clinical variables and echocardiography data between groups using analysis of variance (ANOVA) for continuous variables, and chi‐squared test for categorical variables. The logistic regression analysis was used to calculate odds ratios (ORs) and their 95% confidence intervals (CIs). We used the binary logistic regression model, and multivariate analysis to evaluate the relationship between ABI categories and LVH. Sequential models were developed. Model 1 was adjusted for sex and age. Model 2 included all model 1 variables plus the BMI, SBP, DBP, serum glucose, triglyceride, cholesterol, high‐density lipoprotein cholesterol (HDL‐C), low‐density lipoprotein cholesterol (LDL‐C), blood urea nitrogen (BUN), history of stroke, CAD, and diabetes mellitus (DM). The differences were considered significant if a 2‐tailed *p* value < .05.

## 3 RESULTS

### 3.1 Clinical characteristics of patients by left ventricular hypertrophy

In the whole group of 3953 hypertension patients, there were 1646 patients with LVH. The prevalence of LVH was 41.64%. The clinical characteristics of the study population by LVH are described in Table 1. The average age of patients with LVH was 58.99±7.88 years, and it was 57.43±8.54 years in people without LVH. Males comprised 30.5% of LVH group and 37.3% of the non‐LVH group. Compared to patients without LVH, those with LVH had higher age, weight, BMI, brachial SBP, DBP, triglyceride, HDL‐C, BUN, ABI, and lower height. Moreover, patients with LVH were more frequently women, had a longer history of hypertension and a higher morbidity of CAD and stroke, this may lead to a higher use rate of antiplatelet drugs in LVH group than in non‐LVH group. There was no significant difference in BSA, heart rate, or the prevalence of DM between two groups.

**TABLE 1:** characteristics of participants by LVH

Variables	LVH (*n* = 1646)	Non‐LVH (*n* = 2307)	*p* value
Age (year)	58.99 ± 7.88	57.43 ± 8.54	<.001
Male	502 (30.5%)	861 (37.3%)	<.001
Height (cm)	156.10 ± 7.62	159.09 ± 8.04	<.001
Weight (Kg)	66.45 ± 11.79	64.53 ± 10.91	<.001
BSA (m²)	1.65 ± 0.18	1.64 ± 0.17	.269
BMI (kg/m²)	27.22 ± 4.16	25.43 ± 3.48	<.001
Brachial SBP (mm Hg)	168.26 ± 25.10	159.70 ± 22.26	<.001
Brachial DBP (mm Hg)	97.98 ± 13.38	96.23 ± 11.41	<.001
Heart rate	72.33 ± 12.14	72.70 ± 12.25	.346
Glucose (mmol/L)	5.48 ± 1.53	5.58 ± 1.63	.057
Triglyceride (mmol/L)	1.74 ± 1.27	1.63 ± 1.16	.004
Cholesterol (mmol/L)	5.51 ± 1.06	5.53 ± 1.07	.589
HDL‐C (mmol/L)	1.52 ± 0.32	1.57 ± 0.34	<.001
LDL‐C (mmol/L)	3.16 ± 0.85	3.14 ± 0.83	.443
BUN (mmol/L)	5.58 ± 1.86	5.40 ± 1.69	.002
ABI	1.13 ± 0.11	1.11 ± 0.11	<.001
History of stroke	211 (12.8%)	203 (8.8%)	<.001
History of CAD	193 (11.7%)	168 (7.3%)	<.001
Diabetes mellitus	89 (5.4%)	98 (4.2%)	.095
Duration of hypertension	7.65 ± 7.87	6.19 ± 6.80	<.001
Anti‐platelet drug	209 (12.7%)	232 (10.1%)	.010

*Abbreviations*: BMI, body mass index; BSA, body surface area; BUN, blood urea nitrogen; CAD, coronary artery disease; DBP, diastolic blood pressure; HDL‐C, high‐density lipoprotein cholesterol; LDL, low‐density lipoprotein cholesterol; SBP, systolic blood pressure.

### 3.2 Clinical characteristics and echocardiography data of patients by ankle brachial index

Baseline characteristics of the patients according to the ABI categorical are presented in Table 2. The 3953 hypertensive patients with normal ABI were divided into four groups according to ABI quartile, percentage of males was 29.8%, 30.5%, 35.3%, 42.4%, respectively. Compared with patients with the lowest quartiles of ABI, those with higher quartile of ABI have a higher height, BSA, BUN, LVIDD, IVSd, PWTd, LVM, LVMI, and lower brachial SBP, DBP. There were no significant differences in age, heart rate, RWT, or the prevalence of CAD, stroke, and DM among those groups. Specially, compared with patients with the lowest quartile of ABI, the prevalence of LVH was higher for patients with higher quartile of ABI, the prevalence of LVH in patients among those groups was 37.2%, 38.2%, 45.5%, 45.7%, respectively.

**TABLE 2:** Clinical characteristics and echocardiographic data of participants by ABI

Variables	0.9 < ABI≤1.03	1.04≤ABI≤1.11	1.12≤ABI≤1.20	1.21≤ABI < 1.40	*p* value
Age (year)	57.83 ± 8.67	57.96 ± 8.34	58.25 ± 8.17	58.27 ± 8.10	.547
Male	295 (29.8%)	302 (30.5%)	347 (35.3%)	419 (42.4%)	<.001
Height (cm)	157.00 ± 7.79	157.14 ± 7.52	157.66 ± 8.46	158.98 ± 8.12	<.001
Weight (Kg)	64.90 ± 10.99	64.78 ± 11.20	64.67 ± 11.16	66.97 ± 11.79	<.001
BSA (m²)	1.64 ± 0.17	1.63 ± 0.17	1.64 ± 0.18	1.67 ± 0.18	<.001
BMI (kg/m²)	26.10 ± 3.86	26.19 ± 3.97	25.97 ± 3.74	26.44 ± 3.94	.049
Brachial SBP (mm Hg)	169.25 ± 24.92	164.77 ± 23.88	162.25 ± 23.24	156.79 ± 21.61	<.001
Brachial DBP (mm Hg)	98.36 ± 12.20	97.34 ± 11.77	96.55 ± 12.25	95.59 ± 12.81	<.001
Ankle SBP (mm Hg)	169.30 ± 24.32	180.40 ± 25.52	191.14 ± 26.46	203.33 ± 27.78	<.001
Glucose (mmol/L)	5.50 ± 1.60	5.50 ± 1.37	5.57 ± 1.72	5.58 ± 1.65	.526
Triglyceride (mmol/L)	1.68 ± 1.12	1.63 ± 0.96	1.69 ± 1.39	1.69 ± 1.32	.688
Cholesterol (mmol/L)	5.56 ± 1.11	5.52 ± 1.02	5.47 ± 1.04	5.52 ± 1.10	.340
HDL‐C (mmol/L)	1.56 ± 0.33	1.56 ± 0.35	1.55 ± 0.34	1.54 ± 0.33	.417
LDL‐C (mmol/L)	3.16 ± 0.84	3.16 ± 0.82	3.11 ± 0.82	3.15 ± 0.88	.463
BUN (mmol/L)	5.38 ± 1.79	5.41 ± 1.58	5.59 ± 1.84	5.54 ± 1.82	.029
Heart rate	72.57 ± 12.85	72.75 ± 12.06	72.46 ± 11.83	72.40 ± 12.07	.926
History of Stroke	99 (10%)	102 (10.3%)	109 (11.1%)	104 (10.5%)	.879
History of CAD	101 (10.2%)	90 (9.1%)	82 (8.3%)	88 (8.9%)	.542
Diabetes mellitus	40 (4.0%)	44 (4.4%)	45 (4.6%)	58 (5.9%)	.250

Echocardiographic data					
LVIDD (mm)	44.96 ± 5.03	45.20 ± 5.05	45.69 ± 5.07	46.34 ± 5.23	<.001
PWTd (mm)	9.62 ± 1.35	9.61 ± 1.30	9.80 ± 1.35	9.94 ± 1.40	<.001
IVSd (mm)	9.83 ± 1.65	9.86 ± 1.52	10.07 ± 1.54	10.22 ± 1.58	<.001
LVM (g)	153.17 ± 43.00	154.67 ± 42.54	161.61 ± 43.35	168.83 ± 47.24	<.001
LVMI_h_ (g/m^2.7^)	44.95 ± 12.42	45.67 ± 12.13	47.32 ± 12.20	48.28 ± 12.97	<.001
LVMI_BSA_	93.44 ± 24.54	94.63 ± 24.19	98.69 ± 24.23	100.73 ± 25.82	<.001
RWT	0.43 ± 0.08	0.43 ± 0.07	0.43 ± 0.08	0.43 ± 0.08	.648
LVH	369 (37.2%)	378 (38.2%)	447 (45.5%)	452 (45.7%)	<.001

*Abbreviations*: BMI, body mass index; BSA, body surface area; BUN, blood urea nitrogen; CAD, coronary artery disease; DBP, diastolic blood pressure; HDL‐C, high‐density lipoprotein cholesterol; IVSd, end‐diastolic interventricular septal thickness; LDL, low‐density lipoprotein cholesterol; LVH, left ventricular hypertrophy.; LVIDD, end‐diastolic LV internal dimension; LVMI_BSA_, left ventricular mass index divided by body mass index; LVMI_h_, left ventricular mass index divided by height^2.7^; PWTd, end‐diastolic posterior wall thickness; RWT, relative wall thickness calculated by 2× PWTd/LVIDD; SBP, systolic blood pressure.

### 3.3 Association between ankle brachial index and left ventricular hypertrophy

We draw a restricted cubic spline (RCS) of ABI and LVH to clarify the linear relationship between them with R, the results showed that the non‐linear test result *p* value was 6093 (*p>*.05), suggesting that the relationship between ABI and LVH was linear (Figure S1). Then we built a logistics regression model to study the correlation between ABI and LVH. Our results suggest that ABI (OR 8.77, 95% CI 4.71–16.33, *p* < .001) is a risk factor of LVH as a continuous variable by using Logistic regression model adjusted for age, sex, SBP, DBP, BMI, serum glucose, triglyceride, cholesterol, HDL‐C, LDL‐C, BUN, the history of stroke, CAD and DM, even though it is within the normal range. Then we divided patients into four groups according to ABI quartile: quartile 1 (0.90 < ABI≤1.03), quartile 2 (1.04≤ABI≤1.11), quartile 3 (1.12≤ABI≤1.20), and quartile 4 (1.21≤ABI < 1.40). Table 3 displays the OR estimates for LVH by ABI groups in unadjusted and adjusted models. Compared with the patients with the lowest quartile of ABI, those with the highest quartile of ABI is significantly associated with the increased LVH risk in the unadjusted model (OR 1.42, 95% CI 1.19–1.70, *p* < .001) and in the model 1 after adjusted sex and age (OR 1.48, 95%CI 1.24–1.78, *p* < .001). The relationship remained significantly after further adjustment for SBP, DBP, BMI, height, weight, serum glucose, triglyceride, cholesterol, HDL‐C, LDL‐C, BUN, the history of stroke, CAD and DM in the model 2 (OR 1.83, 95%CI 1.50–2.24, *p* < .001). Similar results were observed in patients with third quartile of ABI, which had significantly increased LVH risk in unadjusted model (OR 1.41, 95% CI 1.17–1.68, *p<*.001), model 1 (OR 1.43, 95%CI 1.19–1.71, *p* < .001), and model 2 (OR 1.61, 95%CI 1.32–1.96, *p<*.001) compared with those with the lowest quartile of ABI. However, their OR values were lower than patients with the highest quartile of ABI. The association of ABI at second quartile with LVH was nonsignificant, before or after adjustment for several covariates.

**TABLE 3:** Odds ratio for left ventricular hypertrophy by ankle brachial index

	0.90<ABI≤1.03		1.04≤ABI≤1.11		1.12≤ABI≤1.20		1.21≤ABI<1.40		ABI (per SD increase)	
	OR (95%CI)	*p*‐value	OR (95%CI)	*p*‐value	OR (95%CI)	*p*‐value	OR (95%CI)	*p*‐value	OR (95%CI)	*p*‐value
Crude	Reference		1.04 (0.87–1.25)	.664	1.41 (1.17–1.68)	<.001	1.42 (1.19–1.70)	<.001	3.72 (2.13–6.48)	<.001
Model1	Reference		1.04 (0.87–1.25)	.661	1.43 (1.19–1.71)	<.001	1.48(1.24–1.78)	<.001	4.35 (2.48–7.65)	<.001
Model2	Reference		1.10 (0.91–1.35)	.327	1.61 (1.32–1.96)	<.001	1.83 (1.50–2.24)	<.001	8.77 (4.71–16.33)	<.001

Model1: adjusted for age, sex.

Model2: adjusted for age, sex, body mass index, height, weight, systolic blood pressure, diastolic blood pressure, serum glucose, triglyceride, cholesterol, high‐density lipoprotein cholesterol, low‐density lipoprotein cholesterol, blood urea nitrogen, the history of stroke, coronary artery disease, and diabetes mellitus.

*Abbreviations*: 95% CI, 95% confidence interval; OR, odds ratio.

We further conducted a stratification analysis of the correlation between LVH and ABI category, and the results are shown in Table 4. In sex strata, the tendency was similar with all patients, the highest and third quartile of ABI were significantly associated with increased LVH risk after adjustment for several covariates. We performed a stratified analysis of the relationship between LVH and at the age of 60 due to literature reports ABI increasing with age until 60–69 years and decreases thereafter. For less than 60 years, association with LVH was significant in third and highest quartile of ABI patients, both in unadjusted model and multivariate‐adjusted model. For at least 60 years, association with LVH was not significant before and after adjustment for several covariates.

**TABLE 4:** Odds ratio for left ventricular hypertrophy by ankle brachial index in stratification analysis

		1.04≤ABI≤1.11		1.12≤ABI≤1.20		1.21≤ABI < 1.40	
	0.9 < ABI≤1.03	Crude OR (95% CI); P‐value	Adjusted OR (95% CI); P‐value	Crude OR (95% CI); P‐value	Adjusted OR (95% CI); P‐value	Crude OR (95% CI); P‐value	Adjusted OR (95% CI); P‐value
**Sex**							
Male	Reference	1.11 (0.79–1.56); 0.558	1.07 (0.73–1.57); 0.726	1.33 (0.96–1.85); 0.085	1.59 (1.10–2.29); 0.013	1.56 (1.14–2.13); 0.006	2.09 (1.47–2.98); <0.001
Female	Reference	1.02 (0.82–1.26); 0.861	1.10 (0.87–1.39); 0.410	1.49 (1.20–1.86); <0.001	1.60 (1.26–2.03); <0.001	1.44 (1.15–1.80); 0.001	1.67 (1.30–2.14); <0.001
**Years**							
≤60	Reference	1.06 (0.84–1.33); 0.649	1.14 (0.89–1.47); 0.303	1.52 (1.21–1.90); <0.001	1.74 (1.36–2.24); <0.001	1.57 (1.25–1.97); <0.001	2.17 (1.69–2.81); <0.001
>60	Reference	1.03 (0.76–1.39); 0.847	1.03 (0.74–1.42); 0.878	1.23 (0.92–1.66); 0.167	1.32 (0.96–1.83); 0.092	1.19 (0.88–1.60); 0.254	1.31 (0.94–1.83); 0.107
**History of CAD**							
No	Reference	1.07 (0.88–1.30); 0.509	1.13 (0.92–1.39); 0.254	1.41 (1.22–1.78); <0.001	1.70 (1.38–2.09); <0.001	1.54 (1.27–1.86); <0.001	2.03 (1.64–2.51); <0.001
Yes	Reference	0.92 (0.52–1.63); 0.770	1.01 (0.53–1.90); 0.983	1.08 (0.60–1.94); 0.800	1.14 (0.59–2.21); 0.697	0.73 (0.41–1.30); 0.290	0.79 (0.41–1.54); 0.487
**History of DM**							
No	Reference	1.05 (0.87–1.27); 0.626	1.11 (0.91–1.36); 0.311	1.42 (1.18–1.71); <0.001	1.67 (1.36–2.04); <0.001	1.38 (1.15–1.66); 0.001	1.80 (1.46–2.22); <0.001
Yes	Reference	0.95 (0.48–1.90); 0.893	0.88 (0.40–1.92); 0.739	1.23 (0.63–2.42); 0.546	1.07 (0.49–2.33); 0.874	1.85 (0.97–3.54); 0.062	1.87 (0.87–4.02); 0.109

Adjusted: age, sex, body mass index, height, weight, systolic blood pressure, diastolic blood pressure, serum glucose, triglyceride, cholesterol, high‐density lipoprotein cholesterol, low‐density lipoprotein cholesterol, blood urea nitrogen, the history of stroke, coronary artery disease, and diabetes mellitus.

*Abbreviations*: 95% CI, 95% confidence interval; OR, odds ratio.

Because ABI associated with arteriosclerotic disease, the relationships between ABI and LVH were examined separately (CAD group and non‐CAD group). For non‐CAD group, the association with LVH was significant in third and highest quartile of ABI groups, both in unadjusted model and multivariate‐adjusted model. However, in CAD group, OR for LVH was not significant for patients with a higher ABI in all models. We also performed a subgroup analysis for diabetes and the results were similar to those for CAD, that is, in the non‐diabetic group, the association between the third and highest of ABI and LVH was significant. However, in the diabetes group, there was no significant association between LVH and patients with higher ABI in all models.

## 4 DISCUSSION

In this study, we observed a strong cross‐sectional association of normal high ABI and increased LVH risk in Chinese hypertensive patients that have a high risk for developing cardiovascular diseases. We found a graded relationship between higher ABI in normal range and increased LVH morbidity which was determined by echocardiography, and this correlation was not affected when adjusted for sex, age, and traditional CVD risk factors. Our results indicated that the higher ABI within the normal range could not only be used as a predictor of asymptomatic organ damage caused by hypertension, but also provided better discrimination for patients with high risk who had traditional risk factors because it could further identify the patients at very high risk of LVH in hypertension groups.

LVH is one of the most common hypertension‐mediated organ damages and is an independent predictor of cardiovascular morbidity and mortality.^16^, ^17^ A meta‐analysis of 30 studies involving a total of 37 700 participants showed that echocardiography LVH was 36–41% in all hypertension participants, and the result is consistent with our study^18^ . The mechanisms underlying hypertension leading to LVH involve complex interactions between cardiomyocytes and non‐cardiomyocytes, including mechanical stretching that activates intracellular signaling cascades,^19^ adaptive immune system imbalance,^20^ and sympathetic nervous system upregulation.^21^ In addition, arterial stiffness is also a risk factor for LVH.^22^ In a stiff arterial tree, the arterial pulse travels faster through the aorta, so the backward wave to reach the ascending aorta during systolic rather than diastolic. This change leads to an increase in the systolic and pulse pressure of the aorta, which in turn increases the left ventricular afterload, and ultimately to increased cardiac muscle mass and LVH.^23^, ^24^

The ABI increases with arterial stiffness and age.^25^ A cross‐sectional study based on people living in communities of different races without clinical cardiovascular disease reported a strong correlation between high ABI measurements and higher LVH.^11^ They speculated that the increase in LVM caused by high ABI is due to medial artery calcification (MAC) which is non‐inflammatory and unrelated to atherosclerosis. MAC leads to arterial stiffness and faster diastolic arterial pulse wave reflex, which in turn leads to an increase in chronic left ventricular afterload. In addition, a recent Japanese study of 13 396 community people with normal ABI (0.9–1.4) found that a high ABI in the normal range was significantly associated with ECG‐determined LVH, suggesting that this may reflect arterial stiffness or increased wave reflexes due to MAC.^15^ Previous animal studies back this up.^26^, ^27^, ^28^ High vitamin D and nicotine induced calcium overload in rats, the model showing extensive MAC without fibrosis or any wall stress changes.^29^ In this model, extracellular calcium‐binding proteins and heterotopic apatite are deposited on the medial elastomer fibers, causing elastomer calcification and ultimately leading to arterial stiffness and compensatory LVH. But the critical value of ABI for predicting MAC induced LVH still needs further study. It has been reported that ABI > 1.30 has a high specificity (99%) and positive predictive value (93%) of MAC discovered by X‐ray, but its sensitivity is only 14%.^30^ In addition, some studies have suggested that an ABI of 1.1–1.3 can represent early MAC,^31^ it is consistent with the research of Hidekazu and coworkers Therefore, ABI≥1.10 may be used as an index for screening asymptomatic LVH caused by MAC in healthy people. Hypertension is an independent risk factor for LVH, but the study of the relationship between normal ABI and LVH had a few reported in hypertensive patients in the past. Our research fills this gap. We found that there was a significant correlation between normal higher ABI (1.12–1.39) and LVH in people with hypertension. In hypertensive population, the incidence of LVH in each group was significantly higher than that in community population, and the OR values for LVH is significantly increased when the ABI is analyzed as a continuous variable. Our results combined with other reports show that a high ABI (1.12–1.39) within the normal range may serve as marker of MAC to further identify patients at high risk for LVH in hypertensive population, thus, distinguishing the very high‐risk groups that are already in the traditional risk factors.

In the stratified analysis, we found that the significant association between normal high ABI and LVH was observed in male, female, less than 60 years, non‐diabetic group and without CAD group, but this correlation was rendered no longer statistically significant in people with at least 60 years, diabetic group, and CAD group. Previous studies have considered that ABI is an important index to predict cardiovascular events such as acute myocardial infarction, heart failure with decreased ejection fraction, and total mortality.^6^, ^32^, ^33^ Most of the participants in the study are at high risk of cardiovascular disease, but there is no known cardiovascular disease. In the cross‐sectional study, it has been reported that ABI can not only identify the high‐risk population of CAD in a large number of people with cardiovascular risk factors,^34^ but also predict the degree of CAD. The more coronary arteries involved, the lower the ABI value.^35^ Therefore, we speculate that the ABI, as a marker of subclinical atherosclerosis, may be affected by atherosclerosis in the patients with CAD, so it can not accurately reflect the degree of arterial stiffness caused by MAC. In addition, the association between ABI and LVH was significantly different between diabetic and non‐diabetic groups. The association between MAC and ABI was reported in 185 patients with diabetes, MAC is most likely present in people with an ABI greater than 1.3, but it is also present in patients with an ABI less than 1.3, making ABI somewhat uncertain in these patients.^30^ Therefore, the disappearance of the correlation between ABI and LVH in the diabetic group may be due to the influence of confounding factors, leading to defects in the diagnosis of ABI.^36^, ^37^

Our results show that in the group under 60 years old, there is a significant correlation between high ABI and LVH in the normal range, and the average age of different groups of ABI groups is increasing. However, in the group over 60 years old, there was no significant difference in the average age of ABI among different groups, and the association with LVH was not significant before and after adjustment for several covariates. Previous studies have shown that aging induces degradation and breakdown of elastic fibers^38^, ^39^ and non‐enzymatic cross‐linking between collagen fibers. The latter change leads not only to an age‐related increase in arterial stiffness, but also to an age‐related increase in blood pressure and vascular lumen dilation, which may lead to an increase in vascular wall stress. Aging is also associated with endothelial dysfunction, decreased NO bioavailability, increased ROS bioavailability, and low inflammation,^40^ changes that are involved in atherosclerosis. A Japanese study of healthy communities shows that ABI may increase with age due to arterial stiffness before the age of 60–69, and then will decrease after 70 years old due to atherosclerosis leading to flow‐limiting arterial stenosis in the lower extremities.^25^, ^41^ Therefore, in hypertension patients over 60 years old, ABI may be influenced by both increased arterial stiffness and atherosclerosis with age, and therefore, ABI may not be a predictor of the risk of LVH due to arterial stiffness in older people with hypertension.

The incidence of cardiovascular events and mortality in patients with LVH are significantly higher than those in the normal population. Hypertension is the most common risk factor and complication of LVH. Currently, there are 245 million people with hypertension in China, so early identification of the high‐risk groups of LVH among hypertension patients and early intervention are of vital importance. Our study observed that a high ABI within the normal range can further identify the extremely high‐risk group of LVH in the hypertension group, providing a new critical point for the prediction of ABI in hypertension‐induced organ damage. In addition, our study examined a large population of patients with hypertension in the Chinses rural area, which minimizes selection bias. We estimated the LVM by echocardiographic, which is more sensitive and specific than ECG.

There are still several limitations of the study should be considered. First, our study only included the hypertensive population of the Han nationality in rural China, which could not represent the urban population. Second, the proportion of men to women included in the study is uneven, with far fewer men than women, which may be due to a large number of male rural residents leaving home to work in cities. Third, this is a cross‐sectional study, so that we can only observe the correlation between ABI and LVH, but not know the causal relationship between the two. Prospective studies are needed to confirm our conclusions. Finally, we speculate that the correlation between normal high ABI and LVH is due to arterial stiffness or increased wave reflex caused by MAC, but we do not have lower limb X‐ray data and wave reflection index to confirm this conjecture. Therefore, the cut‐off points of ABI we used may only be used as reference index for MAC screening in patients with hypertension.

In conclusions, this study shows that there is a significant association between normal higher ABI (1.12–1.39) and LVH in hypertensive people, but this result is more consistent in relatively young patients (≤60 years) and patients without CAD. There was no significant correlation between ABI and LVH in elderly patients with hypertension or hypertension complicated with CAD. Based on the above results, we believe that high ABI within the normal range may be a marker of arterial stiffness in hypertensive patients and is significantly associated with asymptomatic LVH. Therefore, the determination of ABI should be included in the routine examination of patients with hypertension, so as to help identify the very high‐risk population of LVH in patients with hypertension.

## CONFLICT OF INTEREST

All the authors declared that they have no conflict of interest.

## AUTHOR CONTRIBUTIONS

Jin Sun contributed to data interpretation, critical review of the manuscript, drafting the manuscript and revising the comment of reviewers. Shuxia Wang contributed significantly to data collection, the conception of the study and revising the comment of reviewers. Man Li and Yongkang Su contributed to manuscript preparation, analysis tools and assist with data analysis. Shouyuan Ma and Yan Zhang contributed to manuscript revisions. Anhang Zhang, Shuang Cai, Bokai Cheng and Qiligeer Bao helped with data analysis. Ping Zhu contributed to the conception of the study and helped perform the analysis with constructive discussions.

## Supporting information

Supporting Information
