# Supplementary material for: The high normal ankle brachial index is associated with left ventricular hypertrophy in hypertension patients among the Han Chinese
Source: J Clin Hypertens (Greenwich). 2021 Jul 23;23(9):1758–66. doi: 10.1111/jch.14328 (PMC8678752; doi:10.1111/jch.14328)
Supplement: Supplementary file 1 — Supporting Information [file JCH-23-1758-s001.docx]

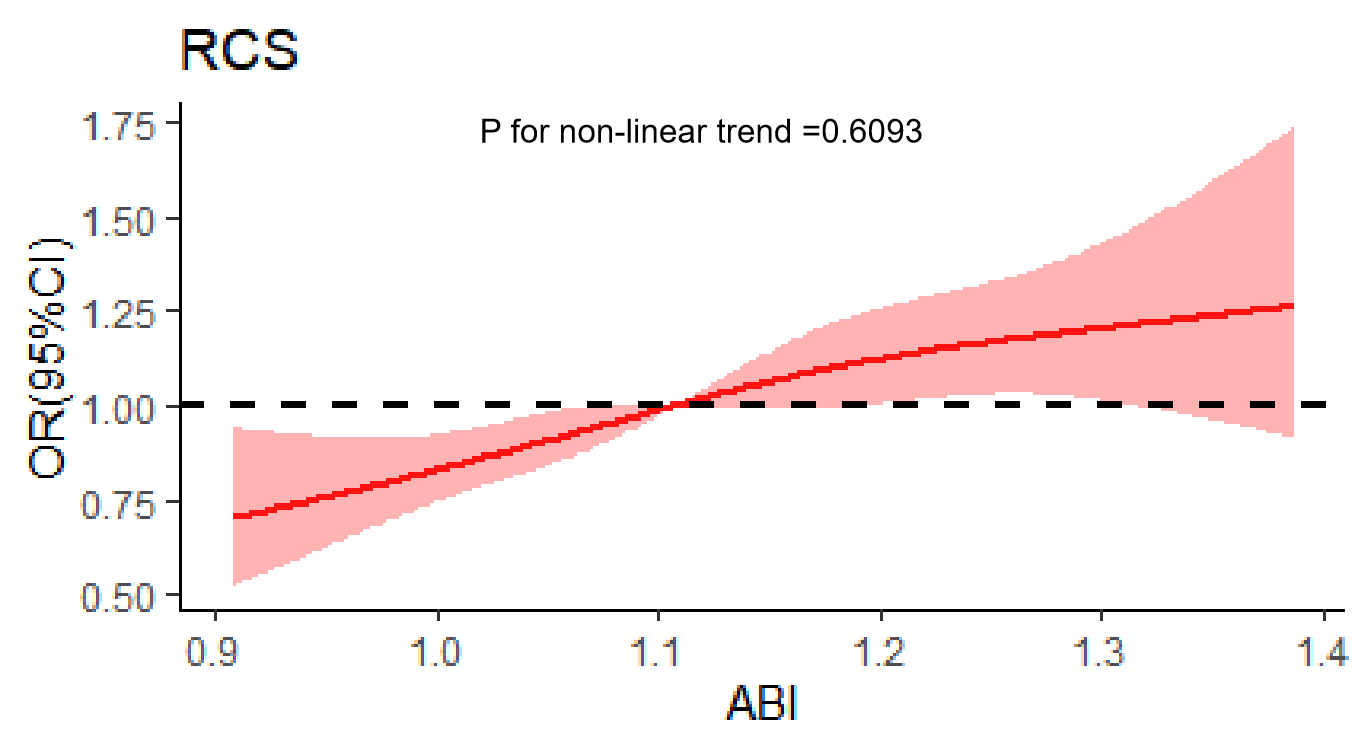


Supplement Figure1 Association between ABI and LVH using a restricted cubic spline regression model.
